# Effect of Prices, Distribution Strategies, and Marketing on Demand for HIV Self-testing in Zimbabwe: A Randomized Clinical Trial

**DOI:** 10.1001/jamanetworkopen.2019.9818

**Published:** 2019-08-28

**Authors:** Wei Chang, Primrose Matambanadzo, Albert Takaruza, Karin Hatzold, Frances M. Cowan, Euphemia Sibanda, Harsha Thirumurthy

**Affiliations:** 1 Department of Health Policy and Management, University of North Carolina at Chapel Hill, Chapel Hill; 2 CeSHHAR Zimbabwe, Avondale, Harare, Zimbabwe; 3 Population Services International, Washington, DC; 4 Department of International Public Health, Liverpool School of Tropical Medicine, Pembroke Place, Liverpool, United Kingdom; 5 Division of Health Policy, Perelman School of Medicine, University of Pennsylvania, Philadelphia; 6 Center for Health Incentives and Behavioral Economics, University of Pennsylvania, Philadelphia

## Abstract

**Importance:**

HIV self-testing is a promising approach for increasing awareness of HIV status in sub-Saharan Africa, particularly in Zimbabwe, where HIV prevalence is 13%. Evidence is lacking, however, on the optimal pricing policies and delivery strategies for maximizing the effect of HIV self-testing.

**Objective:**

To assess demand for HIV self-testing among adults and priority-population subgroups under alternative pricing and distribution strategies.

**Design, Setting, and Participants:**

This randomized clinical trial recruited study participants between February 15, 2018, and April 25, 2018, in urban and rural communities in Zimbabwe. A factorial design was used to randomize participants to a combination of self-test price, distribution site, and promotional message. Individuals and their household members had to be at least 16 years old to be eligible for participation. This intention-to-treat population comprised 3996 participants.

**Interventions:**

Participants were given a voucher that could be redeemed for an HIV self-test within 1 month at varying prices (US $0-$3) and distribution sites (clinics or pharmacies in urban areas, and retail stores or community health workers in rural areas). Vouchers included randomly assigned promotional messages that emphasized the benefits of HIV testing.

**Main Outcomes and Measures:**

Proportion of participants who obtained self-tests in each trial arm, measured by distributor records.

**Results:**

Among the 4000 individuals enrolled, 3996 participants were included. In total, the mean (SD) age was 35 (14.7) years, and most participants (2841 [71.1%]) were female. Self-testing demand was highly price sensitive; 260 participants (32.5%) who were offered free self-tests redeemed their vouchers, compared with 55 participants (6.9%) who were offered self-tests for US $0.50 (odds ratio [OR], 0.14; 95% CI, 0.10-0.19), a reduction in demand of more than 25 percentage points. Demand was below 3% in the $1, $2, and $3 groups, which was statistically significantly lower than the demand in the free distribution group: in pooled analyses, demand was considerably lower among participants in higher-than-$0 price groups compared with the free distribution group (2.8% vs 32.5%; OR, 0.05; 95% CI, 0.04-0.07). In urban areas, demand was statistically significantly higher with pharmacy-based distribution compared with clinic-based distribution (6.8% vs 2.9%; adjusted OR, 2.78; 95% CI, 1.74-4.45). Price sensitivity was statistically significantly higher among rural residents, men, and those who had never received testing before. Promotional messages did not influence demand.

**Conclusions and Relevance:**

This study found that demand for HIV self-testing in Zimbabwe was highly price sensitive, suggesting that free distribution may be essential for promoting testing among high-priority population groups; additionally, pharmacy-based distribution was preferable to clinic-based distribution in urban areas.

**Trial Registration:**

ClinicalTrials.gov identifier: NCT03559959

## Key Points

**Question:**

How is the demand for HIV self-testing influenced by pricing and distribution strategies?

**Findings:**

In a randomized clinical trial of 4000 adults in Zimbabwe, demand for HIV self-testing declined substantially from 32.5% among those offered self-administered tests for free to 6.9% among those offered the tests for US $0.50 and below 3% at prices of US $1 or greater. Price sensitivity was higher among rural residents, men, and those who had never had an HIV test; in urban areas, demand was higher with pharmacy- than clinic-based distribution.

**Meaning:**

This study suggests that demand for HIV self-testing is highly price sensitive in low-income settings; free distribution of self-tests may help promote their use in high-priority population segments.

## Teaser

This randomized clinical trial evaluates the effectiveness of various pricing and distribution methods for a self-administered test for HIV diagnosis in Zimbabwe.

## Introduction

Greater awareness of HIV status and more frequent testing in high-risk populations are essential for realizing the promise of treatment as prevention and achieving the 90-90-90 targets of the Joint United Nations Programme on HIV/AIDS (that by 2020, 90% of people living with HIV will know their HIV status, 90% of people with diagnosed HIV will be on antiretroviral therapy [ART], and 90% of people receiving ART will be virally suppressed).^1^ Yet in sub-Saharan Africa, nearly 20% of people living with HIV were unaware of their status in 2017.^2^ Despite the scale-up of clinic- and community-based models for providing HIV testing services, testing coverage remains suboptimal, particularly among men and other key populations.^3^ To close the testing gap and advance HIV prevention objectives, innovative approaches are needed to increase the uptake of HIV testing in sub-Saharan Africa.

A self-administered test for HIV allows individuals to collect their own sample and to perform a simple, rapid HIV antibody test in the absence of a health care practitioner.^4^ Several oral fluid-based or blood-based HIV tests have received prequalification from the World Health Organization and showed high sensitivity and specificity among lay users.^4^ Existing research shows high interest in and acceptability of HIV self-testing across a wide range of populations.^5,6,7,8,9,10,11,12^ After the 2016 World Health Organization guidelines that recommended large-scale implementation of HIV self-testing, self-tests are becoming more widely available in governmental health facilities and retail outlets in several countries in sub-Saharan Africa with high HIV prevalence.^4^

Donor agencies and governments have heavily subsidized HIV self-tests for distribution in some countries, and private sector availability is emerging in parallel.^13^ However, the cost of self-tests and the price for consumers represent important obstacles to large-scale implementation of HIV self-testing. As countries seek to scale up HIV self-testing for priority populations, little evidence exists on the effect of alternative pricing and marketing strategies on self-testing demand. A growing body of evidence from low-income countries shows that demand for prevention technologies, such as antimalarial bed nets and water filtration solutions, is highly price sensitive.^14,15,16,17,18,19^ Knowing the self-testing demand at various prices in the general population and key subgroups is important for setting appropriate subsidy levels for these self-tests and for understanding the demand for HIV prevention technologies in general. Moreover, with HIV self-testing, information is limited about the optimal distribution approaches for reaching untested individuals and messaging strategies for promoting the adoption of such new technologies. Estimating how demand is affected not only by prices but also by various distribution approaches and types of information provided to consumers can further inform HIV self-testing scale-up efforts.

We conducted a large community-based randomized clinical trial to examine the optimal pricing policies and distribution strategies for HIV self-testing in Zimbabwe.

## Methods

### Study Design, Setting, and Participants

Ethics approval for this randomized clinical trial was obtained from the Medical Research Council of Zimbabwe, the Liverpool School of Tropical Medicine, the University of Pennsylvania, and the University of North Carolina at Chapel Hill. Written informed consent was obtained from individuals who met eligibility criteria and agreed to participate. This trial followed the Consolidated Standards of Reporting Trials (CONSORT) reporting guideline.^20^ The trial protocol is included in Supplement 1.

This randomized clinical trial assessed demand for HIV self-testing among participants who were given vouchers to obtain self-tests at varying prices. After providing written informed consent, participants completed a brief questionnaire. Participants and their household members then received vouchers that specified a randomly selected price and distribution point at which self-tests could be obtained. Some vouchers also included messages designed to promote HIV testing. Redemption of vouchers for self-tests was monitored during the following month.

The study was conducted in rural and urban communities in Zimbabwe, where adult HIV prevalence is 13%.^21^ Communities included a rural area in Mashonaland East Province and a high-density urban area in Mashonaland Central Province. Population Services International Zimbabwe, which provides HIV testing and counseling services in the study communities and leads a national HIV self-testing program, was the main implementation partner for this study.

Between February 15, 2018, and April 25, 2018, research assistants recruited study participants by visiting a random sample of households in study communities. In households that consented to screening, research assistants listed all eligible household members and randomly selected 1 member using a computer-generated randomization scheme. Eligible participants were 16 years of age or older, owned a mobile phone or had access to a household member’s mobile phone, and planned to stay in the study area for at least 12 months. This intention-to-treat population comprised 3996 participants.

### Randomization Procedures

Research assistants gave participants a sealed envelope containing a voucher that revealed the study group assignment to both the participant and the research assistant. The price, distribution point, and promotional message with each voucher were determined using a computer-generated randomization scheme. A factorial design was used to form study groups, with vouchers containing 1 of 5 prices, 2 distribution points, and 4 messaging strategies (Figure). Randomization was stratified by urban and rural sites.

**Figure.**
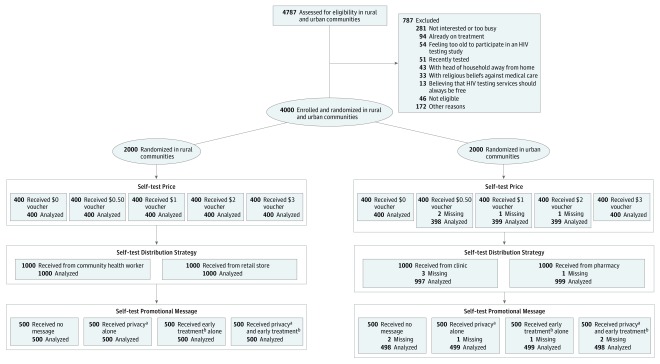
CONSORT Flow Diagram An equal number of individuals from each price group were randomized to each distribution strategy and subsequently to each promotional message type. ^a^Privacy message: “Be the first to know your status and take the right action.” ^b^Early treatment message: “Positive or negative, life is full of hope. If you test HIV-positive, you can immediately access treatment and continue to lead a healthy life.”

### Study Interventions

Participants received a brief verbal description of HIV self-testing during enrollment. The vouchers enabled them to obtain an oral, fluid-based HIV test (OraQuick Rapid HIV-1/2 Antibody Test; OraSure Technologies) in their community within 1 month. Research assistants explained how to redeem the vouchers for self-tests and wrote the expiration date on the vouchers. Although the study’s primary objective was to assess demand among participants, vouchers were also given to participants’ household members aged 16 years or older so that they could obtain self-tests as well. Household members were not required to be present to receive vouchers. Vouchers for each participant and their household members had the same price, distributor, and promotional message. Each voucher included a unique number that could be linked to an individual in the participant’s household.

The out-of-pocket price (in US dollars) the participants had to pay for a self-test ranged from $0 (full subsidy) to $3 (partial subsidy), with 3 intermediate prices of $0.50, $1, and $2. These prices were selected after consultations with key stakeholders in Zimbabwe and after consideration of prevailing retail prices that were $3 or higher.

In rural communities, self-tests were distributed through 48 community health workers and 22 retail stores. In urban communities, self-tests were made available at 8 pharmacies and 12 government clinics. All distributors were equipped with sufficient tests to meet demand from participants.

Two messages to promote HIV testing were included in the vouchers (Figure). One message highlighted the greater privacy and confidentiality of HIV self-testing.^22^ Another message sought to motivate testing by emphasizing that immediate HIV treatment was available for those who received a reactive result. This message was designed to mitigate the perceived burden of living with HIV.^23^ Messages were translated to the Shona language and pretested. Vouchers were randomly assigned to carry 1 of 4 messaging strategies: no message, privacy message, early treatment message, or both messages.

Distributors were asked to collect all vouchers brought by participants and to enter voucher numbers on paper forms and computer tablets. Distributors were compensated $30 at the end of the study for their efforts. They were also monitored every 2 weeks to ensure their fidelity to study procedures. Research assistants verified whether voucher redemptions were reconciled with the inventory of self-tests. Distributors received an additional $5 per compliant monitoring visit, with payment of up to $20 if compliance was assured at 4 monitoring visits.

Self-tests included easy-to-use instructions that had been developed in previous HIV self-testing studies in Zimbabwe.^22^ In addition, distributors were asked to show participants a brief HIV self-testing instructional video on a tablet computer at the time of voucher redemption.

### Data Collection and Study Outcomes

Baseline questionnaire data were collected from participants using ACASI (Audio Computer-Assisted Self-Interview Software; Tufts University) on a tablet computer with headphones. The questionnaire asked for self-reported demographic characteristics, previous HIV testing history, and sexual behavior details.

Uptake of HIV self-testing was monitored at the distribution sites where vouchers were redeemed for self-tests. The distribution sites collected the vouchers, dispensed the tests at the specified prices, and recorded the transaction date on the voucher. Voucher numbers were used to link self-testing uptake to individual participants. The primary prespecified outcome was self-testing demand among participants, defined as a binary indicator of whether a participant obtained a self-test within 1 month. A secondary outcome was self-testing demand among participants’ household members.

### Statistical Analysis

To estimate the effect of price on demand, we used in the primary analysis unadjusted and adjusted logistic regression models to compare demand in each of the 4 nonzero price groups with demand in the free distribution group. The stratification factor of rural compared with urban residence was controlled for in all models, and participants’ sex was included in adjusted models. Logistic regression models adjusting for price and participants’ sex were used to estimate the effect of distribution strategies and promotional messages on self-testing demand. For promotional messages, we compared demand for HIV self-testing in the no message group with that in the privacy alone, early treatment alone, and privacy plus early treatment message groups.

In the secondary analyses, we compared demand for HIV self-testing between the combination of the higher-than-$0 price groups and the free distribution group. Subgroup analyses were performed to determine the effects of nonzero prices by prespecified socioeconomic characteristics and behavioral factors, including income, HIV testing history, number of sexual partners, and condom use. In addition, we performed post hoc subgroup analyses on the basis of residence (urban vs rural), sex, and age according to the policy relevance of studying demand in these particular subgroups. To test whether price sensitivity varied by demographic subgroups, we included a subgroup-price interaction term in linear probability models. We chose linear models because logistic regression models do not provide an odds ratio (OR) interpretation for interaction terms, and the SEs of these interaction terms are not equal to those of average marginal effects in the linear models.^24,25^ In addition to the prespecified main analysis, we conducted post hoc analyses to evaluate the effect of prices on demand among participants’ household members.

All statistical tests were 2-sided, and statistical significance was set at *P* < .05. For the analyses of household members, SEs were clustered at the household level to account for the correlation within a household. Analyses were performed using Stata, version 15.1 (StataCorp LLC).

Power calculations focused on estimating the price sensitivity of self-testing demand. Given limited past data, we assumed 15% of participants would obtain self-tests at the highest price. A sample of 270 participants per price group was required to have 80% power to detect a difference of 10 percentage points or higher in demand between price groups (α = .05). With 2000 participants in rural sites and 2000 in urban sites, the final selection of 5 price points at each site resulted in a sample of 400 participants per price group and 80% power to detect between-group differences in demand of 8 percentage points or higher.

## Results

Among the 4787 individuals assessed for eligibility, 4000 (83.6%) were enrolled and randomized (Figure). Major reasons for nonenrollment included not interested or too busy (281 individuals), already receiving treatment (94), feeling too old to participate in an HIV testing study (54), and having recently been tested (51). Four participants were excluded from analyses because of missing questionnaire data, resulting in an analytical sample of 3996 participants.

Participants in the 5 price groups had largely similar characteristics except for sex (Table 1). Among the 3996 participants, the mean (SD) age was 35 (14.7) years, most (2841 [71.1%]) were female, and 2568 (65.7%) were married. Most participants (3237 [81.7%]) reported having been tested for HIV at least once in the past, but only 1813 (45.4%) reported testing in the past 12 months. Among those who had ever been tested, 259 (8.2%) self-reported being HIV positive. Most participants (2836 [71.0%]) reported having a regular sexual partner, and 178 (5.0%) reported more than 1 sexual partner in the past month. Among those with at least 1 sexual partner in the past month, most participants (1948 [86.6%]) reported consistent condom use.

**Table 1.** Participant Characteristics

Variable	No. of Participants	No. (%)						*P* Value
		Full Sample	Price Group, US $					
			0	0.50	1	2	3	
No.	3996	3996	800	798	799	799	800	
Age, mean (SD), y	3996	35.1 (14.7)	35.0 (14.6)	34.8 (15.1)	35.3 (14.7)	35.5 (14.6)	35.2 (14.7)	.89
Monthly income, median (IQR), US $	1951	60.0 (20.0-150.0)	55.0 (20.0-150.0)	50.0 (20.0-140.0)	60.0 (20.0-175.0)	60.0 (22.0-150.0)	50.0 (20.0-150.0)	.80
Female	3996	2841 (71.1)	573 (71.6)	597 (74.8)	577 (72.2)	543 (68.0)	551 (68.9)	.02
Married	3909	2568 (65.7)	524 (66.9)	526 (67.0)	529 (67.6)	491 (63.2)	498 (63.7)	.20
Educational level	3996							.77
No or some primary schooling		409 (10.2)	79 (9.9)	85 (10.7)	84 (10.5)	90 (11.3)	71 (8.9)	
Completed primary schooling		419 (10.5)	85 (10.6)	78 (9.8)	81 (10.1)	88 (11.0)	87 (10.9)	
Some secondary schooling		857 (21.4)	174 (21.8)	174 (21.8)	175 (21.9)	160 (20.0)	174 (21.8)	
Completed O level		1971 (49.3)	391 (48.9)	408 (51.1)	382 (47.8)	385 (48.2)	405 (50.6)	
Completed A level or higher		340 (8.5)	71 (8.9)	53 (6.6)	77 (9.6)	76 (9.5)	63 (7.9)	
Site	3996							>.99
Rural		2000 (50.1)	400 (50.0)	400 (50.1)	400 (50.1)	400 (50.1)	400 (50.0)	
Urban		1996 (49.9)	400 (50.0)	398 (49.9)	399 (49.9)	399 (49.9)	400 (50.0)	
Ever been tested for HIV	3960	3237 (81.7)	645 (81.9)	654 (82.2)	652 (82.6)	640 (80.8)	646 (81.3)	.89
Tested in the past 12 mo	3996	1813 (45.4)	371 (46.4)	370 (46.4)	347 (43.4)	355 (44.4)	370 (46.2)	.66
Had HIV-positive result in most recent HIV test	3156	259 (8.2)	59 (9.4)	50 (7.8)	49 (7.7)	56 (8.9)	45 (7.2)	.59
Have a regular sexual partner	3996	2836 (71.0)	577 (72.1)	579 (72.6)	581 (72.7)	540 (67.6)	559 (69.9)	.11
Partner ever been tested for HIV	2484	2128 (85.7)	420 (84.5)	443 (86.5)	432 (84.9)	409 (85.0)	424 (87.4)	.65
Partner had HIV-positive result in most recent HIV test	2058	155 (7.5)	31 (7.7)	30 (7.0)	30 (7.3)	33 (8.2)	31 (7.6)	.98
Age at first sexual intercourse, median (IQR), y	2528	20.0 (18.0-22.0)	20.0 (18.0-22.0)	20.0 (18.0-22.0)	20.0 (18.0-23.0)	19.0 (18.0-23.0)	20.0 (18.0-22.0)	.16
No. of partners in past mo	3557							.82
0		1333 (37.5)	267 (37.3)	255 (36.7)	258 (36.0)	286 (39.6)	267 (37.8)	
1		2046 (57.5)	414 (57.8)	409 (58.8)	415 (58.0)	405 (56.0)	403 (57.0)	
>1		178 (5.0)	35 (4.9)	31 (4.5)	43 (6.0)	32 (4.4)	37 (5.2)	
Always used condom in past month (among those with ≥1 partner)	2250	1948 (86.6)	395 (87.4)	389 (87.4)	395 (85.1)	384 (86.5)	385 (86.5)	.85

Abbreviation: IQR, interquartile range.

### Price Sensitivity of Demand

Self-testing demand was higher at rural sites compared with urban sites, but in both communities, demand was sensitive to price (Table 2; eFigure in Supplement 2). Overall, 260 participants (32.5%) who were offered free self-tests redeemed their vouchers, whereas 55 (6.9%) of those offered self-tests for $0.50 redeemed their vouchers (OR, 0.14; 95% CI, 0.10-0.19), a reduction in demand of more than 25 percentage points (Table 2). Demand was also statistically significantly lower in the $1, $2, and $3 groups compared with the free distribution group. In adjusted logistic regression analysis, demand remained associated with price. Results were similar after excluding participants who reported having previously had HIV-positive results.

**Table 2.** Demand for HIV Self-testing by Price

Variable	Full Sample				Rural		Urban	
	Participants, No.	Obtained Self-test, No. (%)	OR (95% CI)		Obtained Self-test, %	AOR (95% CI)^c^	Obtained Self-test, %	AOR (95% CI)^c^
			Unadjusted^a^	Adjusted^b^				
Price group, US $								
0 (free)	800	260 (32.5)	1 [Reference]	1 [Reference]	47.3	1 [Reference]	17.8	1 [Reference]
0.50	798	55 (6.9)	0.14 (0.10-0.19)	0.14 (0.10-0.19)	9.5	0.12 (0.08-0.17)	4.3	0.21 (0.12-0.36)
1	799	21 (2.6)	0.05 (0.03-0.08)	0.05 (0.03-0.08)	4.0	0.05 (0.03-0.08)	1.3	0.06 (0.02-0.15)
2	799	9 (1.1)	0.02 (0.01-0.04)	0.02 (0.01-0.04)	2.0	0.02 (0.01-0.05)	0.3	0.01 (0-0.08)
3	800	6 (0.8)	0.01 (0.01-0.03)	0.01 (0.01-0.03)	1.0	0.01 (0-0.03)	0.5	0.02 (0.01-0.09)
Site								
Rural	2000	255 (12.8)	1 [Reference]	1 [Reference]	NA	NA	NA	NA
Urban	1996	96 (4.8)	0.27 (0.21-0.36)	0.28 (0.21-0.36)	NA	NA	NA	NA
Sex								
Male	1155	116 (10.0)	NA	1 [Reference]	12.8	1 [Reference]	6.0	1 [Reference]
Female	2841	235 (8.3)	NA	0.84 (0.64-1.11)	12.7	0.94 (0.68-1.31)	4.4	0.65 (0.40-1.05)
Pooled price group, US $								
0 (free)	800	260 (32.5)	1 [Reference]	1 [Reference]	47.3	1 [Reference]	17.8	1 [Reference]
>0	3196	91 (2.8)	0.05 (0.04-0.07)	0.05 (0.04-0.07)	4.1	0.05 (0.04-0.07)	1.6	0.07 (0.05-0.12)
Site								
Rural	2000	255 (12.8)	1 [Reference]	1 [Reference]	NA	NA	NA	NA
Urban	1996	96 (4.8)	0.27 (0.21-0.36)	0.28 (0.21-0.37)	NA	NA	NA	NA
Sex								
Male	1155	116 (10.0)	NA	1 [Reference]	12.8	1 [Reference]	6.0	1 [Reference]
Female	2841	235 (8.3)	NA	0.88 (0.67-1.15)	12.7	0.98 (0.71-1.35)	4.4	0.68 (0.42-1.10)

Abbreviations: AOR, adjusted odds ratio; NA, not applicable; OR, odds ratio.

^a^ Results are from a logistic regression model that adjusted for the stratification factor only (ie, rural or urban site).

^b^ Results are from a logistic regression model that adjusted for the stratification factor and any covariate not balanced at baseline (ie, sex).

^c^ Results are from a logistic regression model that adjusted for any covariate not balanced at baseline (ie, sex).

Demand was considerably lower in the combined higher-than-$0 price groups compared with the free distribution group (2.8% vs 32.5%; OR, 0.05; 95% CI, 0.04-0.07). When examining rural and urban sites separately, we observed that demand remained associated with price. In rural sites, demand was highest in the free distribution group (47.3%) and statistically significantly lower at higher prices, with 9.5% of participants obtaining self-tests in the $0.50 group (adjusted OR [AOR], 0.12; 95% CI, 0.08-0.17) and 4.0% obtaining self-tests in the $1 group (AOR, 0.05; 95% CI, 0.03-0.08). In urban sites, 17.8% of participants in the free distribution group redeemed vouchers, and demand also declined statistically significantly at higher prices.

Demand among 4923 household members was also highly sensitive to price in logistic regression models that adjusted for sex and age (eTable 1 in Supplement 2). Effect sizes were similar to those found among study participants.

### Demand by Distribution Strategy and Promotional Message

In rural sites, demand was higher with community health worker distribution compared with retail store distribution (14.0% vs 11.7%), but this difference was not statistically significant in logistic regression analyses that adjusted for self-test price offered to participants and sex of participants (AOR, 0.77; 95% CI, 0.56-1.05) (Table 3). In urban areas, demand was statistically significantly higher with pharmacy-based distribution compared with clinic-based distribution (6.8% vs 2.9%; AOR, 2.78; 95% CI, 1.74-4.45). Demand was also higher with pharmacy-based distribution among participants who were offered the free vouchers (eTable 2 in Supplement 2).

**Table 3.** Demand for HIV Self-testing by Distribution Strategy and Promotional Message

Groups	No. of Participants	Obtained Self-test, No. (%)	AOR (95% CI)^a^	
			Adjusted for Price	Adjusted for Price and Sex
Group by distribution strategy				
Rural				
CHW	1000	138 (14.0)	1 [Reference]	1 [Reference]
Retail store	1000	117 (11.7)	0.77 (0.56-1.05)	0.77 (0.57-1.05)
Urban				
Clinic	997	28 (2.9)	1 [Reference]	1 [Reference]
Pharmacy	999	68 (6.8)	2.78 (1.74-4.45)	2.79 (1.74-4.48)
Group by promotional method				
No message	998	102 (10.2)	1 [Reference]	1 [Reference]
Privacy alone^b^	999	85 (8.5)	0.78 (0.56-1.09)	0.79 (0.56-1.11)
Early treatment alone^c^	999	84 (8.4)	0.77 (0.55-1.07)	0.77 (0.55-1.08)
Privacy and early treatment^b^^,^^c^	1000	80 (8.0)	0.72 (0.51-1.01)	0.74 (0.53-1.04)

Abbreviations: AOR, adjusted odds ratio; CHW, community health worker.

^a^ Results are from logistic regression models.

^b^ Privacy message: “Be the first to know your status and take the right action.”

^c^ Early treatment message: “Positive or negative, life is full of hope. If you test HIV-positive, you can immediately access treatment and continue to lead a healthy life.”

Promotional messages included with vouchers did not influence demand. Overall, participants who received vouchers with no added messages to promote HIV testing were most likely to obtain self-tests (10.2%), whereas those with messages promoting the privacy of self-testing, immediate antiretroviral therapy for those obtaining a reactive result, or both had demand between 8.0% and 8.5%. Demand did not differ statistically significantly between these groups, among all participants, or among those offered the free vouchers (Table 3 and eTable 2 in Supplement 2).

### Subgroup Analyses for Price Sensitivity of Demand

Self-testing demand varied considerably among various participant subgroups, but it remained highly sensitive to price in all subgroups (Table 4). Demand declined more steeply with higher prices in rural areas than in urban areas, and the difference in price sensitivity was statistically significant (β = 0.02; SE = 0.01; *P* < .0001). Male participants had higher demand compared with female participants at low prices but not at higher prices, indicating a higher price sensitivity that was statistically significant (β = –0.08; SE = 0.04; *P* = .04). Price sensitivity was also higher among participants 25 years or younger compared with older participants (β = –0.02; SE = 0.01; *P* < .001). Demand and price sensitivity did not differ much between those with below-median incomes compared with those with above-median incomes and similarly between those with low-risk compared with high-risk sexual behaviors. However, owing to the magnitude of missingness in income and number of sexual partners (Table 1), these results should be interpreted with caution. Participants who reported never having had an HIV test displayed greater price sensitivity compared with those who had been tested before (β = 0.01; SE = 0.01; *P* = .03).

**Table 4.** Demand for HIV Self-testing and Price Sensitivity in Participant Subgroups

Variable	Obtained Self-tests, No./Total No. (%)					Effect of Price >US $0^a^		*P* Value for Subgroup-Price Interaction^b^
	US $0 Price Group	US $0.50 Price Group	US $1 Price Group	US $2 Price Group	US $3 Price Group	AOR (95% CI)	*P* Value	
Obtained HIV self-test	260/800 (32.5)	55/798 (6.9)	21/799 (2.6)	9/799 (1.1)	6/800 (0.8)	0.06 (0.05-0.08)	<.001	NA
Urban/rural								
Urban	71/400 (17.8)	17/398 (4.3)	5/399 (1.3)	1/399 (0.3)	2/400 (0.5)	0.07 (0.05-0.12)	<.001	<.001
Rural	189/400 (47.3)	38/400 (9.5)	16/400 (4)	8/400 (2)	4/400 (1)	0.05 (0.04-0.07)	<.001	
Sex								
Female	173/573 (30.2)	34/597 (5.7)	17/577 (2.9)	6/543 (1.1)	5/551 (0.9)	0.06 (0.05-0.09)	<.001	.04
Male	87/227 (38.3)	21/201 (10.4)	4/222 (1.8)	3/256 (1.2)	1/249 (0.4)	0.05 (0.03-0.08)	<.001	
Age, y								
>25	195/549 (35.5)	48/524 (9.2)	17/551 (3.1)	7/564 (1.2)	5/564 (0.9)	0.07 (0.05-0.09)	<.001	<.001
≤25	65/251 (25.9)	7/274 (2.6)	4/248 (1.6)	2/235 (0.9)	1/236 (0.4)	0.04 (0.02-0.07)	<.001	
Income								
≤Median	65/219 (29.7)	12/248 (4.8)	6/203 (3)	4/235 (1.7)	1/225 (0.4)	0.06 (0.04-0.1)	<.001	.22
>Median	55/162 (34)	16/159 (10.1)	3/169 (1.8)	3/173 (1.7)	3/158 (1.9)	0.07 (0.04-0.12)	<.001	
Tested								
Never before	41/143 (28.7)	8/142 (5.6)	2/137 (1.5)	2/152 (1.3)	0/149	0.05 (0.03-0.1)	<.001	.03
Tested before	217/645 (33.6)	47/654 (7.2)	19/652 (2.9)	7/640 (1.1)	6/646 (0.9)	0.06 (0.05-0.08)	<.001	
No. of partners in past mo								
≤1	202/564 (35.8)	37/536 (6.9)	9/550 (1.6)	9/555 (1.6)	5/527 (0.9)	0.05 (0.04-0.07)	<.001	.98
>1	14/46 (30.4)	2/38 (5.3)	3/52 (5.8)	0/41	1/44 (2.3)	0.08 (0.03-0.22)	<.001	
Used condoms in last mo								
Always	150/395 (38)	28/389 (7.2)	7/395 (1.8)	7/384 (1.8)	4/385 (1)	0.05 (0.03-0.07)	<.001	.54
Not always	12/57 (21.1)	4/56 (7.1)	3/69 (4.3)	0/60	0/60	0.11 (0.04-0.3)	<.001	

Abbreviations: AOR, adjusted odds ratio; NA, not applicable.

^a^ Results are from logistic regression models of HIV self-testing demand, with the indicator of the >$0 price group ($0 price group as the reference) and controls for participant sex. Each AOR is for the >$0 price group compared with the free distribution group when the sample is restricted to the specific subgroup defined in the first column.

^b^ *P* value is for the interaction between the subgroup (first row of each subgroup as the reference) and the >$0 price group in linear probability models, which include all observations with nonmissing values for the subgroup variable defined in the first column and control for sex.

## Discussion

In high-HIV-prevalence areas of Zimbabwe in which the need to increase coverage of HIV testing is substantial, demand for HIV self-testing was highly sensitive to price. Compared with offering self-tests for free, charging prices as low as $.50 led to strikingly large reductions in demand, from 32.5% to 6.9%. Demand was even lower at higher prices of $1 to $3. Demand was highly sensitive to price across many population subgroups, but price sensitivity was highest in priority subgroups such as rural residents, men, and those who had never had an HIV test. In addition, pharmacy-based distribution resulted in the highest uptake of self-tests in urban areas, whereas in rural areas, demand did not differ between tests distributed by community health workers and by retail outlets.

This trial provides evidence that can be used to develop optimal pricing policies and distribution strategies for self-testing. Although the market for HIV self-tests is still developing, prices in low- and middle-income countries presently range from $3 to $6 per test in the public sector and are even higher in the private sector.^26^ A recent initiative of the Bill and Melinda Gates Foundation lowered the price of a widely used oral, fluid-based self-test (OraQuick Rapid HIV-1/2 Antibody Test) to $2 in high-prevalence countries.^13^ As HIV self-testing receives consideration as part of intensified HIV elimination efforts, this study’s findings suggest that further subsidies will be needed to achieve desired increases in testing coverage. The higher price sensitivity in priority populations with lower rates of previous testing underscores the need to subsidize self-tests.

This study also contributes to a growing literature that uses field experiments to assess demand for health products and services in low-income settings. Demand for diagnostics and life-saving health technologies is remarkably sensitive to price, even at low prices. The large reduction in demand at prices higher than $0 is consistent with studies showing that small price increases are associated with large declines in demand for antimalarial bed nets, water disinfectant solutions, and treatments for acute diseases.^14,15,16,17,18,19^ To our knowledge, no studies have been conducted on the price sensitivity of self-testing demand using experimental methods and revealed preferences. Studies using stated preferences have shown that prices do influence demand for HIV and malaria testing,^12,27,28,29,30,31^ but a key distinction in the present study is that observed demand at prices higher than $0 was considerably lower than what would be expected given the stated willingness to pay in other studies conducted in eastern and southern Africa. The differences between study populations may explain this observation, but it is plausible that stated willingness to pay exaggerates actual demand.

Demand was not even higher when participants, particularly in urban areas, were offered HIV self-tests free of charge. About half of rural participants and one-fifth of urban participants obtained self-tests when offered to them for free. The higher uptake in rural areas could be the result of lower rates of previous testing and lack of confidential, private, or convenient testing options. In urban areas, in contrast, previous testing rates were higher and people could obtain anonymous testing at nearby locations. Studies of demand for other health products have observed much higher uptake under free distribution,^14,17^ but self-testing demand at similar prices may be lower owing to the psychological distress about obtaining an HIV-positive result and the greater stigma associated with HIV than other diseases such as malaria.^23^

In this study, promotional messages emphasizing the benefits of HIV self-testing did not increase demand. In fact, not including any message with vouchers generated the highest demand among the 4 messaging strategies we tested. The reason for the lack of demand might be that short promotional messages were not salient enough to highlight the positive attributes of self-testing or address the anticipated emotional burdens.^29^ In addition, participants may have already been familiar with the information conveyed in the messages. More than 80% of participants had had an HIV test, and they could have heard about the benefits of early treatment. Future work that tests messages designed to address other perceived limitations of HIV self-testing, such as concerns over pretest and posttest counseling or accuracy of results,^32^ might reveal new ways to promote the uptake of self-testing.

### Limitations

This trial has several limitations. First, most participants were female, because men were less likely to be home during recruitment. However, demand and price sensitivity among household members, who were 57% male, were similar to those found among study participants. Second, demand for HIV self-testing was indicated by voucher redemption, but use of self-tests was not measured. However, studies have shown that people who obtained self-tests also used them, and experiments of demand for other health products have generally refuted the notion that paying for a health product increases the likelihood of use.^14,15,17,33^ Third, we did not collect data on test results and thereby cannot determine whether the yield of individuals with HIV-positive results varied by price or by distribution strategy.^18^ Fourth, we did not assess spillover effects on households. Spillover effects may heighten price sensitivity if individuals in the nonzero price groups chose not to pay for a self-test after knowing that their neighbor received a free voucher. Alternatively, social learning from those who tried the self-tests first may lead to higher uptake in the nonzero price groups. Fifth, we did not assess linkage to care by individuals who received an HIV-positive result, which was beyond the scope of the study but is important for future research to address.

## Conclusions

This study found that demand for HIV self-testing is price sensitive in Zimbabwe, even at relatively low prices and especially in rural areas. These results suggest that free distribution of self-tests is likely to be necessary for increasing HIV testing coverage among high-priority groups, such as men and those who have never had an HIV test.

## Notes

Supplement 1. **Trial Protocol**

Supplement 2. **eFigure.** Demand for HIV Self-Tests By Price, Overall, and By Site **eTable 1.** Demand for HIV Self-Tests By Price Among Household Members of Study Participants **eTable 2.** Demand for HIV Self-Tests By Distribution Strategy and Promotional Message Among Those in the Free Voucher Arm

Supplement 3. **Data Sharing Statement**
