# Supplementary material for: Effect of Prices, Distribution Strategies, and Marketing on Demand for HIV Self-testing in Zimbabwe: A Randomized Clinical Trial
Source: JAMA Netw Open. 2019 Aug 28;2(8):e199818. doi: 10.1001/jamanetworkopen.2019.9818 (PMC6716290; doi:10.1001/jamanetworkopen.2019.9818)
Supplement: Supplement 1. — Trial Protocol [file jamanetwopen-2-e199818-s001.pdf]

A Field Experiment to Assess the Demand for HIV Self-Tests in Zimbabwe

Protocol Version: Version 1.6

Protocol Date: 10 November 2017

Principal Investigator: **Professor Frances M. Cowan**

STUDY TEAM

Frances M Cowan, PhD
CeSHHAR Zimbabwe &
Liverpool School of Tropical Medicine
Email: frances.cowan@lstmed.ac.uk

Harsha Thirumurthy, PhD
University of Pennsylvania,
Philadelphia, PA, USA
Email: hthirumu@upenn.edu

Euphemia Sibanda, PhD
CeSHHAR Zimbabwe
Harare, Zimbabwe
Email: euphemia@ceshhar.co.zw

Karin Hatzold, MD
Population Services International
Harare, Zimbabwe
Email: khatzold@psi.org

Sudhanshu Handa, PhD
University of North Carolina at Chapel Hill
Chapel Hill, NC, USA
Email: shanda@email.unc.edu

Wei Chang, MPH
University of North Carolina at Chapel Hill
Chapel Hill, NC, USA
Email: wei@unc.edu

ACRONYMS

ACASI	Audio Computer-Assisted Self-Interivew
ART	Antiretroviral therapy
BBST	Blood-based self-test
CeSHHAR	Centre for Sexual Health and HIV/AIDS Research
HIVST	HIV self-testing
MRCZ	Medical Research Council of Zimbabwea
PSI	Population Services International
RDT	Rapid diagnostic test
SMS	Short Message Service
SRA	Stringent Regulatory Authority
SSA	Sub Saharan Africa
STAR	Self-Testing in Africa
UNAIDS	Joint United Nations Programme on HIV/AIDS
WHO	World Health Organization

BACKGROUND

Although there are over 25 million people living with HIV in Sub-Saharan Africa (SSA), about half of them are unaware of their status. Universal HIV testing uptake is therefore a critical first step towards ending the HIV/AIDS epidemic and represents the largest obstacle to meeting the first of the UNAIDS 90-90-90 targets – that is, by 2020, 90% of all people living with HIV should know their HIV status.[1,2] Certain populations are disproportionately vulnerable and at elevated risks of HIV infection, including young women and adolescent girls as well as key populations such as sex workers and men who have sex with men.[3] However, current HIV testing services approaches do not reach everyone, most often either because services are not accessible to them or because people are concerned about lack of privacy and fear of stigma and discrimination.[4] Consequently, many people living with HIV only present at health care facilities late in their infection when they are already ill, reducing their chance of successful treatment.[5]

HIV self-testing is a promising approach to increase HIV testing uptake. The WHO defines HIV self-testing (HIVST) as a specific process in which a person collects his or her specimen (oral fluid or blood), performs a test, and interprets the result, often in private. Self-tests can circumvent a number of structural and health systems barriers to uptake of HIV testing. With the development of simple, oral fluid-based tests that have achieved high sensitivity and specificity, a number of countries in SSA are developing policies to implement and support self-testing and the WHO has recently called for the scale-up of HIVST.[6] Research conducted in a number of different countries, including Zimbabwe, shows high acceptability of and interest in self-testing across a wide range of populations as well as high accuracy in the hands of lay users.[7-12] By having the potential to reach first-time testers and non-frequent testers, individuals with undiagnosed HIV, and those at ongoing risk who need frequent retesting, HIVST is a promising and innovative approach to increase HIV testing uptake. As countries consider alternative ways to scale-up HIVST, it is essential to know the demand for self-test kits in the general population and in high-risk segments of the population. In particular, knowing what the demand for self-tests might be at various prices is important for developing optimal pricing strategies – and subsidy levels – for the test kits. It is also vital to understand the implications of self-test pricing policies for the demand that can be expected by high-risk individuals who do not typically access other HIV testing modalities.

Novel rapid diagnostic tests for HIV self-testing are available, but evidence on its effectiveness and implementation strategies in low-resource settings is lacking. There are currently three HIVST products, one oral fluid-based and two blood-based rapid diagnostic tests (RDTs), that are approved by a Stringent Regulatory Authority (SRA), but all of these are in high income countries: Oraquick In-Home HIV Test (Orasure Technologies USA), an oral fluid RDT, is available in the United States; blood-based RDTs are available in France (Autotest VIH, AAZ Labs, France) and the United Kingdom (BioSure HIV Self-Test, Biosure Ltd., United Kingdom). Only one of the current RDTs for HIVST is WHO prequalified, which facilitates registration in resource-limited countries in SSA. In addition, at least six blood-based RDTs and three oral fluid-based RDTs for HIVST are currently under consideration or in development. The majority of these products are intended for low and middle-income markets, including SSA, with most manufacturers expecting demand in these target markets to be coming from the public sector (e.g. governments and international donors).

Because HIV self-tests have the potential to be a game changer in increasing HIV testing uptake, there is considerable interest in what strategies are optimal for distributing self-tests and what prices (if any) should be charged for self-tests. As countries consider alternative ways to scale-up HIVST, it is essential to know the demand for self-test kits in the general population and in high-risk segments of

the population. In particular, knowing what the demand for self-tests might be at various prices is important for developing optimal pricing strategies – and subsidy levels – for the test kits. It is also vital to understand the implications of self-test pricing policies for the demand that can be expected by high-risk individuals who do not typically access other HIV testing modalities. Generating real world evidence on the public health and subsidy implications of different pricing policies for self-tests can provide the information that donor organizations and country governments need for making important decisions.

A field experiment can address the evidence gap and inform policy decisions regarding the optimal pricing policies and distribution strategies for HIV self-testing. To fill the evidence gap and inform policy decisions, we propose to conduct a field experiment to obtain "revealed preference" estimates of the likelihood that individuals will purchase oral fluid-based and blood-based HIV self-tests at alternative prices. Such an approach is useful for estimating the price sensitivity (or elasticity) of demand for self-tests. Economists have conducted field experiments to determine the demand for various essential healthcare products in many countries including Kenya, Zambia, and Malawi. These products include antimalarial bed nets, medical male circumcision services, and chlorine-based water treatment products – the results of these studies have been informative in policy debates about whether it is preferable to charge for essential healthcare products (partial government subsidy) or offer them for free instead (full subsidy).[13-15] The proposed field experiment for HIV self-tests will also estimate the extent to which demand for self-tests differs by certain distribution channels such as retail outlets, public sector health facilities, and other community-based venues. Importantly, the experiment will also test whether the provision of HIV self-tests can be targeted more cost-effectively to reach high-risk persons and non-recent testers by examining the demand for self-tests in these populations in responses to various prices and distribution strategies. Last, with additional funding in the future, we will explore whether demand for repeated HIV self-testing is contingent on the price offered initially. The results of the study will provide vital information required by policy makers and implementers on the optical pricing and distribution strategies for introducing HIV self-tests and scaling up HIVST in resource limited settings.

SPECIFIC AIMS

The specific aims of the proposed study are as follows:

- Aim 1: Estimate demand for oral fluid-based and blood-based HIV self-tests at different out-of-pocket prices and determine the sensitivity of demand to various distribution strategies.
- Aim 2: Determine whether there are differences in HIV testing history and HIV risk profile of individuals who purchase or obtain HIV self-tests at low vs. high prices.
- Aim 3: Examine whether individuals' demand for repeat self-test purchases is contingent on initial prices (with additional funding in the future).

STUDY DESIGN

Overview of Study Design

We will conduct a rigorous field experiment in urban and rural communities near Harare, Zimbabwe, to estimate individuals' demand for oral fluid-based and blood-based HIV self-tests. About 4,000 adults will be randomly selected in study communities, administered a short questionnaire, and given referral vouchers that will enable them to obtain HIV self-tests at randomly allocated prices and distribution sites. All participants will be given a brief description of HIVST during the time of enrollment. In addition, all self-tests will include easy-to-use instructions that have been carefully developed based on prior studies in Zimbabwe. A link to an instructional video may also be provided to those who have access to a

phone and express interest in receiving a link to the video. Uptake of HIVST will be monitored at the distribution sites, where the vouchers will be collected in exchange for self-tests; the voucher numbers will enable us to link HIVST uptake to individual participants.

In a subsequent study with additional funding, we will also seek to determine the long-term implications of offering self-tests at very low prices initially; specifically we will assess whether free distribution initially suppresses individuals' willingness to pay for self-tests in the future. Roughly 12 months after the initial voucher offer, participants who provided a mobile phone number they can access within their household will be contacted via phone-based text messages and offered the same HIV self-tests again at one common non-zero price. The purchases at 12 months will once again be monitored and linked to participants via a unique identifier that is included in the text messages.

In Aim 1, we will generate estimates of the price sensitivity of demand for self-tests and also determine how the demand varies by distribution channels. In Aim 2, we will perform sub-group analyses to test whether key segments of the population are different in their likelihood of purchasing self-tests at various prices and distribution channels. This will indicate whether HIV self-tests can be targeted more cost-effectively to priority groups, such as those who do not typically use facility-based testing services. In Aim 3, we will explore to what extent individuals' demand for repeated self-testing is contingent on the price that is initially offered to them. This is a relevant question to understand whether HIV self-tests can facilitate repeated testing among high-risk individuals and whether offering HIV self-tests for free or at low price initially has long-term implications for the prices that can be charged. Separately, we will explore whether promotion messages on the vouchers have an effect of HIVST uptake.

Study Locations
The study will be conducted in collaboration between MoHCC, PSI/Zimbabwe and the Centre for Sexual Health and HIV/AIDS Research (CeSHHAR) Zimbabwe. The proposed study will take place in two locations: 1) Seke Rural, a rural district in Mashonaland East province, and 2) Bindura Urban, a high density area in Mashonaland Central.

Eligibility Criteria
Individuals who meet with study staff will be screened for the following eligibility criteria:

- Age 16 years or older
- Willing and able to provide written informed consent for participation
- Intends to stay in the study area for at least 12 months

Consent and Enrollment Processes
Households in the study locations will be randomly selected using a door-to-door strategy. In each household, research assistants will randomly select one eligible individual and invite them to participate in the study, following a standard randomization protocol. Research assistants will review the informed consent form with eligible individuals. Those who provide consent will be enrolled into the study and administered a brief baseline questionnaire.

Randomization Procedures
After completing the baseline questionnaire, participants will be given general information about HIVST and then assigned to one of the experimental arms based on computer-generated randomization. Participants will be given one referral voucher that offers a self-test kit at the random price and distribution site determined on the tablet computer. The vouchers will be valid for one month after the enrollment date – meaning that individuals will have up to one month to decide whether to

purchase/obtain self-tests. Each voucher can be used to redeem one self-test kit at the specified price and distribution site. The other household members above 16 years or age will also be offered one voucher per person. Vouchers for the same household will have the same price, distributor, and promotion messages.

Research assistants will clearly explain to participants how they can redeem the voucher. The out-of-pocket price that participants must pay for self-tests will be written clearly on the voucher and will range from $0 (free/fully subsidized) to up to $3. Up to five different prices will be used in the study and all of the prices will be below the current full market value of self-tests, meaning that all participants will be offered self-tests at a discounted price. Each voucher will have a unique identifier to determine whether the participants obtained self-tests within one month at the distribution sites. Computer-generated block randomization will be used to determine the sequence of prices & distribution sites on vouchers.

Distribution channels at which the self-tests will be sold or offered will be determined in consultation with PSI/Z and CeSHHAR staff. In rural communities, the distribution channels may include community health workers and retail shops to ensure access for all participants. In urban areas, the distribution channels may include pharmacies and local clinics.

Roughly 12 months after the enrollment ends, participants who own a cell phone will be offered the same HIV self-tests at one common price through short message service (SMS). This offer will be made by text message containing a unique number, and participants will be told they can purchase up to two self-tests by showing the text message to the staff at various distribution sites within one month. A unique identifier will be included in each text message and recorded at the time of sale in order to determine which participants purchased self-tests.

Data Collection Activities

Baseline data. At the time of enrollment, participants will be administered a baseline questionnaire to record information on demographic characteristics, contact information, prior HIV testing history, and self-reported sexual behavior. Data will be recorded on encrypted, password-protected tablet computers. Participants will then be assigned to one of the experimental arms based on computer-generated randomization that is performed on the tablet computers. The result of the randomization will be recorded by research assistants, including the price, distribution site, and the unique identifier on each voucher.

Demand for HIV self-tests. To purchase oral fluid-based or blood-based HIV self-tests at subsidized prices with the paper voucher, participants must present the voucher in person within a month at the designated distribution site and pay the price specified on the voucher. The distribution sites will collect the vouchers, dispense HIV self-tests at the specified prices, and record the date of the transaction on the voucher.

Demand for repeat testing. To purchase HIV self-tests during the repeat testing portion of the study, participants must show the text message to the staff at designated distribution sites and pay the common price that will be established. Using a pre-distributed list of all cellphone numbers that were included in the study or an electronic system where cell phone numbers can be entered and confirmed, the distribution sites will verify that the participant is eligible to purchase self-tests and then dispense self-tests at the common price. The unique number included in the text message will be recorded so that it is possible to determine which participants purchased self-tests.

Study staff will undergo comprehensive training on the study design and methods, and we will also work closely throughout the study with the distribution sites to make sure they understand the purpose of the study and can perform relevant tasks. We will conduct weekly phone calls with designated staff at each distribution site to answer questions and resolve issues. Research assistants will collect the paper vouchers from the distribution sites approximately one month after enrollment ends and similarly will collect the redemption records one month after the text-message offer.

Outcomes
The primary outcome will be the proportion of individuals that purchased or obtained at least one HIV self-test in each of the study groups. This will enable us to estimate the proportion of individuals who would buy self-tests at various prices, and the proportion of individuals who would buy self-tests at various combinations of price and distribution site.

In addition, we will examine how the likelihood of purchase vary by price and distribution site in key segments of the population, including individuals who have and have not tested for HIV in the past, individuals with low and high risk of HIV infection based on self-reported sexual behavior, and individuals with low and high income.

Finally, with additional funding in the future, we will compare the proportions of individuals who purchase HIV self-tests at the common price offered at about 12 months based on whether they initially received a partially or fully subsidized price. This will enable us to explore whether individuals' demand for HIV self-tests over the long term is contingent on the price that is initially offered to them.

Statistical Analyses
We will perform chi-squared tests to compare uptake at various non-zero prices to uptake at the price of zero (i.e. free distribution). We will also use regression analyses to estimate the elasticity (i.e. price-sensitivity) of the demand for self-tests. We will estimate unadjusted regression models as well as adjusted regression modes that include covariates such as individual characteristics, particularly those that differ between study groups. The subgroup analyses will be performed by comparison of means and by regression analyses that use interaction terms. Robust standard errors that allow for clustering of standard errors within communities and households will be reported for all regressions.

Sample Size and Power Calculation
The power analysis for this study focused on the primary outcome that compares the proportion of individuals who purchased at least one HIV self-test with vouchers at low vs. high price points. The power calculations allow for analyses to be stratified by rural vs. urban clusters. Assuming 15% of individuals would make a purchase when offered the highest price, the sample size required to detect a 10-percentage-point increase in the percentage of individuals who purchase at lower prices will be 270 per group (80% power and a type I error of 0.05). Thus, a sample size of about 2,000 adults in rural areas and 2,000 adults in urban areas (for a total sample of about 4,000 adults) will allow for five different price points to be used in each area.

Data Management
CeSHHAR Zimbabwe will be the data coordinating centre. This unit is headed by an experienced data manager. Data will be stored securely on password-protected servers (for electronic data) and locked cabinets (in the case of consent forms). Deidentified data will be shared with other study team members for analyses.

Questionnaire data will be collected using Audio Computer-Assisted Self-Interview Software (ACASI) on a tablet with headphones attached. Data validity checks will be built into the ACASI platform. In the field, data will be backed up daily onto "cloud storage". Laptop computers on which any data may be stored will be kept in locked storage at all times. Field teams will download data into a password protected database accessible only to the project data manager and named study personnel, on a central computer. This will be backed up daily. Hard-copy data will be stored in participant files and locked in a file cabinet located in a secure room accessible only to key study personnel. Participants will be asked to provide written informed consent for participation.

Timeline

	2017									*2018*			
**Activities**	**May**	**Jun**	**Jul**	**Aug**	**Sep**	**Oct**	**Nov**	**Dec**	**…**	Jul	Aug	Sep	Oct
Protocol and SOP preparation, IRB submission													
Hire & train staff; Select distribution channels													
Enroll study participants; Distribute paper voucher													
Collect data on purchase of HIV self-tests with paper voucher													
Analyze data and prepare manuscripts for Aim 1 & 2									…				
<u>SUBSEQUENT STUDY</u> Offer HIV self-tests with SMS voucher													
Collect data on purchase of HIV serf-tests with SMS voucher													
Analyze data and prepare manuscripts for Aim 3													

Dissemination of Research Findings
Research findings will be presented promptly after study completion in various venues, including meetings with government and donors, at scientific conferences, and in peer-reviewed journals. Dissemination of evidence from research undertaken through this investment will also leverage the existing research dissemination plan developed by the UNITAID/PSI HIV Self-Testing Africa (STAR) project. Results will be shared at local country level in Zimbabwe through HIVST technical working groups that have been put in place and are chaired by the government. STAR consortium partners, London School of Hygiene and Tropical Medicine, WHO and PSI have established HIVST and STAR technical web-sites where updates and outcomes are shared on regular basis as soon as new evidence is available. Research findings will be discussed with members of the STAR Technical Advisory Group, which brings together public health experts, scientists and policy makers to guide research and program implementation as well as with the HIVST technical working group established by WHO.

Ethical considerations
This protocol will be subject to review and approval by institutional review boards at all participating institutions, including the Medical Research Council of Zimbabwe (MRCZ) and the Liverpool School of Tropical Medicine. This will include, according to each IRB's requirements, approval prior to the initiation of research, on-going adverse event monitoring, periodic review, and final study reporting.

Participant Informed Consent and Remuneration

Informed consent will be required from all research participants. Participants will be provided with information about the research and study procedures by a trained interviewer, and will have the opportunity to ask questions as part of the informed consent process.

Participants will not receive financial compensation for participating in the experiment given the benefit of purchasing HIV self-tests at prices below the market value. Moreover, providing compensation to participants will undermine the objective of measuring the real-world demand for HIV self-tests in the population.

Distribution sites will be reimbursed for their efforts in kit distribution and data collection, in the form of a flat rate (for community health workers) or a flat rate plus a bonus based on data quality/kit stock accuracy (for retail shops and pharmacies). We may also perform random audits to verify that test kits are being offered at prices indicated on the vouchers.

Confidentiality

The research team will ensure that all research data collected are numbered with a unique ID and not named. Only phone number will be collected from the participants. Questionnaire data and purchase outcomes will be linked using the unique identifier printed on each voucher or sent through SMS messages.

All study staff will undergo Good Clinical Practice and ethics training. All people working with CeSHHAR Zimbabwe sign a confidentiality agreement; they have very strict confidentiality procedures in place.

Study Modification and Discontinuation

The study may be modified or discontinued at any time by the IRBs as part of their duties to ensure that research participants are protected.

Protocol Deviations and Exceptions

The investigator should not implement any deviation from, or changes of, the protocol without prior and documented approval from the Ethical Committee of an amendment, except where necessary to eliminate an immediate hazard(s) to trial subjects. The investigator should document and explain any deviation from the approved protocol and to file waivers received from MRCZ, if applicable.

References

1. UNAIDS. *Global AIDS Update.* Geneva, Switzerland: Joint United Nations Programme on HIV/AIDS;2016.
2. UNAIDS. *90-90-90: An ambitious treatment target to help end the AIDS epidemic.* UNAIDS;2014.
3. UNAIDS. *Gap Report.* Geneva, Switzerland: UNAIDS;2014.
4. Musheke M, Ntalasha H, Gari S, et al. A systematic review of qualitative findings on factors enabling and deterring uptake of HIV testing in Sub-Saharan Africa. *BMC Public Health.* 2013;13:220.
5. Siedner MJ, Ng CK, Bassett IV, Katz IT, Bangsberg DR, Tsai AC. Trends in CD4 count at presentation to care and treatment initiation in sub-Saharan Africa, 2002-2013: a meta-analysis. *Clinical infectious diseases : an official publication of the Infectious Diseases Society of America.* 2015;60(7):1120-1127.
6. World Health Organization. *Guidelines on HIV self-testing and partner notification.* Geneva: World Health Organization;2016.
7. Napierala Mavedzenge S, Baggaley R, Corbett EL. A review of self-testing for HIV: research and policy priorities in a new era of HIV prevention. *Clinical infectious diseases : an official publication of the Infectious Diseases Society of America.* 2013;57(1):126-138.
8. Figueroa C, Johnson C, Verster A, Baggaley R. Attitudes and Acceptability on HIV Self-testing Among Key Populations: A Literature Review. *AIDS Behav.* 2015;19(11):1949-1965.
9. Thirumurthy H, Akello I, Murray K, et al. Acceptability and feasibility of a novel approach to promote HIV testing in sexual and social networks using HIV self-tests. . Paper presented at: International AIDS Society (IAS) conference2015; Vancouver, Canada.
10. Choko AT, Desmond N, Webb EL, et al. The Uptake and Accuracy of Oral Kits for HIV Self-Testing in High HIV Prevalence Setting: A Cross-Sectional Feasibility Study in Blantyre, Malawi. *PLoS medicine.* 2011;8(10):e1001102.
11. Kalibala S, Tun W, Cherutich P, Nganga A, Oweya E, Oluoch P. Factors associated with acceptability of HIV self-testing among health care workers in Kenya. *AIDS Behav.* 2014;18 Suppl 4:S405-414.
12. Johnson C, Baggaley R, Forsythe S, et al. Realizing the potential for HIV self-testing. *AIDS Behav.* 2014;18 Suppl 4:S391-395.
13. Dupas P. Short-run subsidies and long-run adoption of new health products: Evidence from a field experiment. *Econometrica.* 2014;82(1):197-228.
14. Cohen J, Dupas P. Free distribution or cost-sharing? Evidence from a randomized malaria prevention experiment. *The Quarterly Journal of Economics.* 2010:1-45.
15. Ashraf N, Berry J, Shapiro JM. Can higher prices stimulate product use? Evidence from a field experiment in Zambia. *The American economic review.* 2010;100(5):2383-2413.
