# Supplementary material for: Effect of Prices, Distribution Strategies, and Marketing on Demand for HIV Self-testing in Zimbabwe: A Randomized Clinical Trial
Source: JAMA Netw Open. 2019 Aug 28;2(8):e199818. doi: 10.1001/jamanetworkopen.2019.9818 (PMC6716290; doi:10.1001/jamanetworkopen.2019.9818)
Supplement: Supplement 2. — eFigure. Demand for HIV Self-Tests By Price, Overall, and By Site eTable 1. Demand for HIV Self-Tests By Price Among Household Members of Study Participants eTable 2. Demand for HIV Self-Tests By Distribution Strategy and Promotional Message Among Those in the Free Voucher Arm [file jamanetwopen-2-e199818-s002.pdf]

Supplementary Online Content

Chang W, Matambanadzo P, Takaruza A, et al. Effect of prices, distribution strategies, and marketing on demand for HIV self-testing in Zimbabwe: a randomized clinical trial. *JAMA Netw Open*. 2019;2(8):e199818. doi:10.1001/jamanetworkopen.2019.9818

eFigure. Demand for HIV Self-Tests By Price, Overall, and By Site

eTable 1. Demand for HIV Self-Tests By Price Among Household Members of Study Participants

eTable 2. Demand for HIV Self-Tests By Distribution Strategy and Promotional Message Among Those in the Free Voucher Arm

This supplementary material has been provided by the authors to give readers additional information about their work.

©2019 Chang W et al. *JAMA Network Open*

eFigure. Demand for HIV Self-Tests By Price, Overall, and By Site

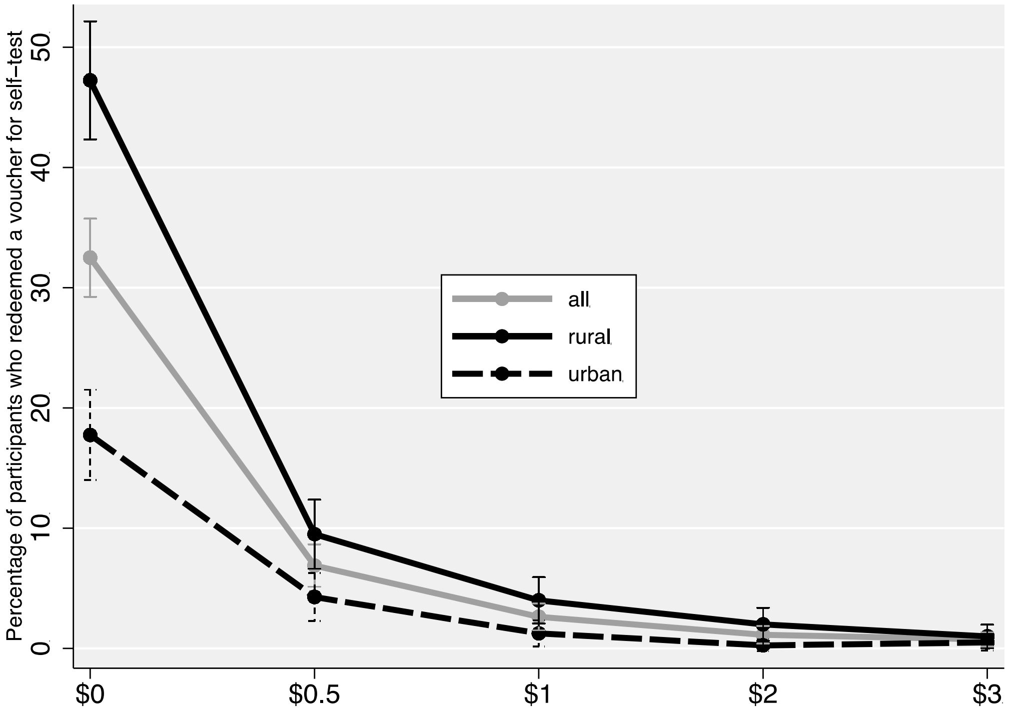

©2019 Chang W et al. *JAMA Network Open*

eTable 1. Demand for HIV Self-Tests By Price Among Household Members of Study Participants

	Full Sample				Rural		Urban	
	**Participants, No.**	**Obtained self-test, No.**	**Obtained self-test, %**	**Adjusted OR (95% CI)[a]**	**Obtained self-test, %**	**Adjusted OR (95% CI)[b]**	**Obtained self-test, %**	**Adjusted OR (95% CI)[b]**
Price Group								
$0 (free)	966	286	29.6%	Reference	45.1%	Reference	12.6%	Reference
$0.5	992	54	5.4%	0.12 (0.08 – 0.19)	6.4%	0.08 (0.05 – 0.14)	4.5%	0.33 (0.17 – 0.64)
$1	1026	21	2.0%	0.04 (0.02 – 0.08)	3.5%	0.04 (0.02 – 0.08)	0.4%	0.03 (0.01 – 0.12)
$2	973	5	0.5%	0.01 (0.00 – 0.03)	1.0%	0.01 (0.01 – 0.03)	0.0%	--
$3	966	3	0.3%	0.01 (0.00 – 0.03)	0.4%	0.00 (0.00 – 0.04)	0.2%	0.01 (0.00 – 0.11)
Site								
Rural	2527	286	11.3%	Reference	--	--	--	--
Urban	2396	83	3.5%	0.23 (0.16 – 0.33)	--	--	--	--
Sex								
Male	2823	204	7.2%	Reference	11.2%	Reference	3.5%	Reference
Female	2100	165	7.9%	1.00 (0.79 – 1.27)	11.5%	0.95 (0.72 – 1.26)	3.5%	1.15 (0.72 – 1.84)
Age								
Age > 25	3138	256	8.2%	Reference	11.9%	Reference	4.2%	Reference
Age ≤ 25	1785	113	6.3%	0.75 (0.59 – 0.97)	10.3%	0.87 (0.64 – 1.17)	2.3%	0.50 (0.29 – 0.84)
Pooled Price								
$0 (free)	966	286	29.6%	Reference	45.1%	Reference	12.6%	Reference
Price>$0	3957	83	2.1%	0.05 (0.03 – 0.06)	2.9%	0.04 (0.02 – 0.05)	1.3%	0.09 (0.05 – 0.17)
Site								

©2019 Chang W et al. *JAMA Network Open*

	Full Sample				Rural		Urban	
	**Participants, No.**	**Obtained self-test, No.**	**Obtained self-test, %**	**Adjusted OR (95% CI)[a]**	**Obtained self-test, %**	**Adjusted OR (95% CI)[b]**	**Obtained self-test, %**	**Adjusted OR (95% CI)[b]**
Rural	2527	286	11.3%	Reference	--	--	--	--
Urban	2396	83	3.5%	0.23 (0.16 – 0.33)	--	--	--	--
Sex								
Male	2823	204	7.2%	Reference	11.2%	Reference	3.5%	Reference
Female	2100	165	7.9%	0.98 (0.77 – 1.24)	11.5%	0.93 (0.7 – 1.23)	3.5%	1.12 (0.70 – 1.79)
Age								
Age > 25	3138	256	8.2%	Reference	11.9%	Reference	4.2%	Reference
Age ≤ 25	1785	113	6.3%	0.75 (0.58 – 0.96)	10.3%	0.86 (0.64 – 1.16)	2.3%	0.51 (0.30 – 0.87)

Abbreviations: OR, odds ratio; CI, confidence interval

[a] Logistic regression model that adjusted for the stratification factor (i.e., rural or urban site) and any covariate not balanced at baseline (i.e., sex and age); standard errors clustered at household level

[b] Logistic regression model that adjusted for any covariate not balanced at baseline (i.e., sex and age); standard errors clustered at household level

©2019 Chang W et al. *JAMA Network Open*

eTable 2. Demand for HIV Self-Tests By Distribution Strategy and Promotional Message Among Those in the Free Voucher Arm

	**Study Group by Distribution Strategy**			
	Rural		Urban	
	CHW	Retail Store	Clinic	Pharmacy
No. of Participants	200	200	200	200
Obtained self-test, No.	100	89	22	49
Obtained self-test, %	50%	45%	11%	25%
OR (95% CI)[a]	Reference	0.8 (0.54 – 1.19)	Reference	2.63 (1.52 – 4.54)
AOR (95% CI), adjusted for sex[a]	Reference	0.8 (0.54 – 1.19)	Reference	2.63 (1.52 – 4.55)
	**Study Group by Promotional Message**			
	No Message	Privacy[b] alone	Early treatment[c] alone	Privacy[b] & Early treatment[c]
No. of Participants	200	200	200	200
Obtained self-test, No.	75	59	65	61
Obtained self-test, %	38%	30%	33%	31%
OR (95% CI) [a]	Reference	0.7 (0.46 – 1.06)	0.8 (0.53 – 1.21)	0.73 (0.48 – 1.11)
AOR (95% CI), adjusted for sex[a]	Reference	0.71 (0.47 – 1.08)	0.81 (0.54 – 1.23)	0.76 (0.5 – 1.16)

Abbreviations: OR, odds ratio; AOR, adjusted odds ratio; 95% CI, confidence interval; CHW, community health worker

[a] Results are from logistic regression models.

[b] "Privacy" message: Be the first to know your status and take the right action.

[c] "Early treatment" message: Positive or negative, life is full of hope. If you test HIV-positive, you can immediately access treatment and continue to lead a healthy life.

©2019 Chang W et al. *JAMA Network Open*
