# Supplementary material for: Effect of Prices, Distribution Strategies, and Marketing on Demand for HIV Self-testing in Zimbabwe: A Randomized Clinical Trial
Source: JAMA Netw Open. 2019 Aug 28;2(8):e199818. doi: 10.1001/jamanetworkopen.2019.9818 (PMC6716290; doi:10.1001/jamanetworkopen.2019.9818)
Supplement: Supplement 3. — Data Sharing Statement [file jamanetwopen-2-e199818-s003.pdf]

Data Sharing Statement

Chang. Effect of Prices, Distribution Strategies, and Marketing on Demand for HIV Self-Testing in Zimbabwe. *JAMA Netw Open*. Published August 28, 2019. 10.1001/jamanetworkopen.2019.9818

Data
Data available: Yes
Data types: Deidentified participant data
How to access data: We plan to make the data available via Dataverse.
When available: With publication

Supporting Documents
Document types: None

Additional Information
Who can access the data: Researchers whose proposed use of the data has been approved.
Types of analyses: For a specified purpose approved by the study team
Mechanisms of data availability: After approval of a proposal
